# 

**Published:** 1871-06

**Authors:** 


LIST OF PAYMENTS FOR THE JOURNAL.—Drs. D. Yarbrough, $1; F. M. Thomason, 50 cts. ; J. B Warren, $1 ; J. C. Turnipseed, $1 ; J. J. A. Smith, $3; John Dickinson, $1; Rudicil and Calhoun, 50; W. A. Childers, 50; J. E. Bell, $1; A. H. S. Harden, $1; D. McCall, $1; W. M. Meador, $1 50; W. R. Bell, $1 50; S. H. Freeman, $3; W. P. Harden, $3; W. W. Fitts, $3; J. W. Taylor, $3; J. P. Taylor, $3; Geo. M. Burdett, $3; H. Perdue, $3; P. M. Tidwell, $3; S. W. Vaughan, $3; G. F. Wirsen, $3; D. C. Bennett, $3; Caldwell & Reed, $3; McDowell & Strother, $3; Paul Faver, $3 ; Dr. Dornall, $3.

